# Anatomical evidence of an indirect pathway for word repetition

**DOI:** 10.1212/WNL.0000000000008746

**Published:** 2020-02-11

**Authors:** Stephanie J. Forkel, Emily Rogalski, Niki Drossinos Sancho, Lucio D'Anna, Pedro Luque Laguna, Jaiashre Sridhar, Flavio Dell'Acqua, Sandra Weintraub, Cynthia Thompson, M.-Marsel Mesulam, Marco Catani

**Affiliations:** From the Departments of Neuroimaging and Forensic and Neurodevelopmental Sciences (S.J.F., N.D.S., L.D., P.L.L., F.D., M.C.), Natbrainlab, Sackler Institute of Translational Neurodevelopment, Institute of Psychiatry, Psychology and Neuroscience, King's College London, UK; Mesulam Center for Cognitive Neurology and Alzheimer's Disease (E.R., J.S., S.W., M.-M.M.), Department of Psychiatry and Behavioral Sciences (E.R.), and Department of Neurology (M.M.M.), Northwestern University Feinberg School of Medicine, Chicago, IL; Division of Neuroscience and Experimental Psychology, School of Biological Sciences (N.D.S., S.W.), University of Manchester, UK; and Neurobiology of Language Recovery, Aphasia and Neurolinguistics Research Laboratory, Communication Sciences and Disorders, and Neurology (C.T.), Northwestern University, Chicago, IL.

## Abstract

**Objective:**

To combine MRI-based cortical morphometry and diffusion white matter tractography to describe the anatomical correlates of repetition deficits in patients with primary progressive aphasia (PPA).

**Methods:**

The traditional anatomical model of language identifies a network for word repetition that includes Wernicke and Broca regions directly connected via the arcuate fasciculus. Recent tractography findings of an indirect pathway between Wernicke and Broca regions suggest a critical role of the inferior parietal lobe for repetition. To test whether repetition deficits are associated with damage to the direct or indirect pathway between both regions, tractography analysis was performed in 30 patients with PPA (64.27 ± 8.51 years) and 22 healthy controls. Cortical volume measurements were also extracted from 8 perisylvian language areas connected by the direct and indirect pathways.

**Results:**

Compared to healthy controls, patients with PPA presented with reduced performance in repetition tasks and increased damage to most of the perisylvian cortical regions and their connections through the indirect pathway. Repetition deficits were prominent in patients with cortical atrophy of the temporo-parietal region with volumetric reductions of the indirect pathway.

**Conclusions:**

The results suggest that in PPA, deficits in repetition are due to damage to the temporo-parietal cortex and its connections to Wernicke and Broca regions. We therefore propose a revised language model that also includes an indirect pathway for repetition, which has important clinical implications for the functional mapping and treatment of neurologic patients.

Repetition deficits are frequently observed in patients with primary progressive aphasia (PPA) in which the progressive degenerative nature of the underlying pathologies causes changes in perisylvian cortical areas and associated pathways without destroying their architecture entirely^1,2^; hence cortical and white matter damage in PPA can be studied in vivo with cortical morphometry and white matter tractography, respectively.

The traditional language model, which includes Broca and Wernicke regions connected by the arcuate fasciculus, predicts that repetition deficits result from a lesion to the arcuate fasciculus disconnecting both language areas. But the disconnection hypothesis of repetition deficits has been challenged on several grounds. There are occasional reports of patients with lesions to the arcuate fasciculus but preserved repetition^3^ or atypical patients with repetition deficits associated with lesions outside the arcuate fasciculus.^4^ These cases suggest that lesions to the arcuate fasciculus may not be sufficient or necessary to cause repetition deficits. In addition, studies in neurosurgical patients report an association between repetition difficulties and stimulation to the cortex of the posterior temporal gyrus or supramarginal gyrus.^5^ Thus, whether repetition deficits are related to a lesion of the arcuate fasciculus or the temporo-parietal cortex remains an open question.

In this study, we used the direct and indirect model of the arcuate fasciculus^6^ to assess whether repetition deficits in PPA can be attributed to purely cortical damage, a disconnection mechanism, or a combination.

## Methods

### Participants

Thirty patients (64.27 ± 8.51 years, 16 female) with PPA and 22 age- and sex-matched controls (62.68 ± 6.14 years, 10 female) were recruited from the Primary Progressive Aphasia Program at the Mesulam Center for Cognitive Neurology and Alzheimer's Disease of the Northwestern University Feinberg School of Medicine. All participants provided written informed consent. The diagnosis of PPA was made by experienced clinicians (M.-M.M., S.W., C.T.) based on isolated and progressive language impairment.^7–9^ In addition to the root diagnosis of PPA, all patients received a descriptive diagnosis of logopenic (n = 6), agrammatic (n = 7), semantic (n = 8), or mixed or severe/unclassified (n = 9) variants of PPA according to established guidelines.^9,10^ We analyzed all PPA subtypes together and, therefore, some of the correlations may reflect between-group differences. This was in part accounted for by replicating the analysis within each subtype.

### Behavioral assessment

The neuropsychological assessment focused on the repetition subtest obtained within the revised Western Aphasia Battery.^11^ The repetition subtest contains a total of 15 items of ascending difficulty ranging from the repetition of single words to word strings, phrases, and sentences (repetition 100). Considering that single word and sentence repetition performance can show double dissociation, suggesting possible different mechanisms underlying these processes,^12^ the analysis was repeated using only the sentence items of the repetition subtest (repetition 66). All items of the test were designed to remain within the working memory span to reduce interferences due to memory and executive deficits.^11^ To account for the potential confounding effect of nonfluent speech on word and sentence repetition, we repeated the analysis using a composite measure derived from a sentence-to-word repetition ratio. This ratio is also likely to be sensitive to the effects of verbal working memory and sequencing impairment. To further characterize the profile of language deficits, performances on other tests were obtained. These included the Peabody Picture Vocabulary Test (PPVT, max score 36) for single word comprehension and the Boston Naming Test (BNT, % correct). Speech samples were recorded from each participant while they told the story of Cinderella from a wordless picture book and speech rate measured as number of words per sentence (Mean Length of Utterance, MLU) and words per minute (WPM). Handedness was estimated with the Edinburgh Handedness Inventory (EHI).

### Diffusion imaging acquisition, data processing, and tract dissections

Diffusion imaging acquisition was carried out at the Center for Translational Imaging at Northwestern University, Chicago. A total of 72 contiguous near-axial slices were acquired for each volume on a 3T Siemens (Munich, Germany) Trio MRI system, using an acquisition sequence fully optimized for clinical tractography, providing isotropic (2 × 2 × 2 mm) resolution and whole head coverage. Sixty diffusion-weighted volumes (b-value of 1,000 s/mm^2^) and 8 volumes without diffusion gradient were acquired.

Tractography preprocessing was performed using ExploreDTI (exploredti.com). Head motion and geometric distortions were corrected simultaneously by reorientating the b-matrix. Remaining outliers due to head motion and cardiac pulsation were excluded using RESTORE. The tensor model was fitted to the data using a nonlinear least square fitting procedure. A whole brain tractography algorithm using Euler integration and the following settings was applied: step size = 0.5 mm, fractional anisotropy threshold ≥0.15, and angle threshold ≤35. Whole brain tractography was exported to TrackVis (trackvis.org) using Startrack software (mr-startrack.com) in MATLAB 2016b (mathworks.com).

TrackVis was used to perform virtual dissections. Regions of interest (ROIs) were defined manually on the axial, coronal, and sagittal fractional anisotropy images of each participant. The dissector (N.D.S.) was trained by an expert tractographer (M.C.) on 10 practice datasets and dissections for this study began after the dissector reached high reliability (i.e., intraclass correlation analysis >0.90). The dissector was blind to the results of the cortical atrophy analysis and the identity of the individual datasets.

**Figure 1:**
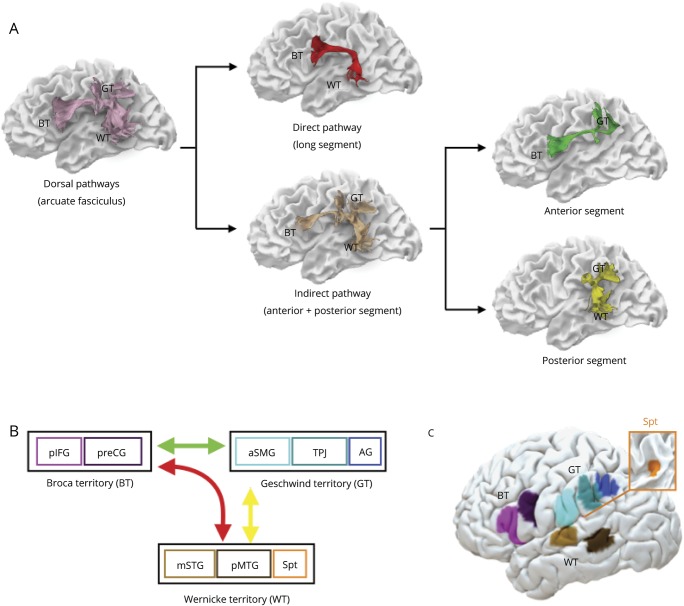
Direct and indirect language networks and cortical regions in the frontal, parietal, and temporal lobes (A) The arcuate fasciculus (lilac) connecting Broca (BT), Geschwind (GT), and Wernicke (WT) territories can be divided into subcomponents. The direct pathway corresponds to the long segment of the arcuate fasciculus (red) connecting temporal and frontal regions. The indirect pathway (beige) is composed of the anterior (green) and posterior (yellow) segments of the arcuate fasciculus connecting temporal and frontal regions via the inferior parietal lobe. Individual cortical terminations of the white matter pathways are indicated on the surface reconstruction in (B) (schematic) and (C) (brain surface). The color coding is chosen to highlight the differences between areas and white matter pathways but has no further implications. AG = angular gyrus; aSMG = anterior supramarginal gyrus; mSTG = middle part of the superior temporal gyrus; pIFG = posterior inferior frontal gyrus; pMTG = posterior middle temporal gyrus; preCG = precentral gyrus; Spt = Sylvian parietal temporal area; TPJ = temporo-parietal junction.

Three ROIs were delineated in the white matter of the frontal (precentral and inferior frontal gyrus), temporal (superior and middle temporal gyrus), and inferior parietal lobe (angular and supramarginal gyrus) according to previously published criteria.^6,13–15^ All streamlines passing through the frontal and temporal ROIs were included in the direct pathway of the arcuate fasciculus (long segment). All streamlines connecting the parietal ROI to either the frontal or the temporal ROI were included in the indirect pathway of the arcuate fasciculus. The indirect pathway was further divided into an anterior segment between the frontal and parietal ROIs and a posterior segment between temporal and parietal ROIs (figure 1A). The size of the direct and indirect pathways of the arcuate fasciculus was estimated by calculating the sum of the volume of all voxels intersected by the streamlines of the long segment and the anterior and posterior segments, respectively. This index provides an estimate of the volume occupied by the streamlines of the tract of interest, which correlates with the degree of white matter atrophy and the severity of clinical symptoms as demonstrated by previous studies in PPA.^16^

### T1-weighted imaging acquisition and cortical gray matter morphometry

T1-weighted magnetization-prepared rapid gradient echo images were acquired using a sequence with the following measures: repetition time = 2,300 ms, echo time = 2.86 ms, flip angle = 9°, field of view = 256 mm, slice thickness = 1 mm. MRIs were processed using FreeSurfer (v5.1.0; surfer.nmr.mgh.harvard.edu/). Cortical thickness estimates were calculated by measuring the distance between representations of the white–gray and pial–CSF boundaries across each point of the cortical surface.^17^ Statistical surface maps were generated to identify participant-specific peak atrophy patterns using a general linear model that displayed differences in cortical thickness for each vertex along surface representations of the entire neocortex between patients with PPA and 35 previously described cognitively healthy controls^18^ using a false discovery rate (FDR) of 0.05.

Cortical morphometry analysis was performed on a total of 8 perisylvian ROIs. These ROIs were selected based on previous neuroimaging studies on repetition in stroke cohorts and PPA^8,22,23^ fMRI activation studies in healthy controls,^19,20^ probability maps of cytoarchitectonic studies,^21^ and cortical projections of the arcuate fasciculus^6,14^ (figure 1, B and C). In the inferior frontal lobe, ROIs were selected in the ventral precentral gyrus and the posterior inferior frontal gyrus.^20^ Both frontal regions receive projections from the direct and indirect pathways of the arcuate fasciculus and are included within the Broca territory. In the inferior parietal lobe, the Geschwind territory encompasses the anterior supramarginal gyrus, the temporo-parietal junction (TPJ; i.e., posterior SMG and posterior superior temporal gyrus), and the angular gyrus. These regions receive projections only from the indirect pathway of the arcuate fasciculus and previous stroke studies indicated that lesions to these regions are associated with repetition deficits.^22,23^ Three temporal regions of interest form Wernicke territory: the Sylvian parietal temporal area (Spt),^24,25^ the posterior part of the middle temporal gyrus, and the middle portion of the superior temporal gyrus. These regions receive projections from both the direct and indirect pathway of the arcuate fasciculus.^6,14^ Montreal Neurological Institute coordinates were converted to Talairach space using mni2tal (sprout022.sprout.yale.edu/mni2tal/mni2tal.html). The ROIs were created by a 10 times dilation of the vertex corresponding to the coordinates in Talairach space.

Normalized volumes for each ROI were calculated using the following equation and used for all analyses:
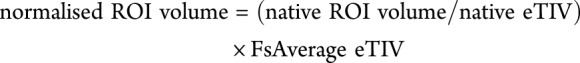
Native ROI volume refers to the participant-specific uncorrected volumes and native eTIV is the participant-specific estimated total intracranial volume. FsAverage eTIV is the estimated total intracranial volume for the FreeSurfer average participant (surfer.nmr.mgh.harvard.edu/fswiki/eTIV; FsAverage eTIV = 1,948,106 mm^3^).

For display purposes, the locations of cortical regions and white matter pathways of a healthy control are shown on the pial and white matter surface, respectively (figure 1). Both templates are available from the authors upon request.

### Statistical analysis

Statistical analyses were performed using IBM SPSS 24 (SPSS Inc., Chicago, IL). Student *t* test or χ^2^ test were used to assess differences between controls and patients. Univariate analysis of variance with familywise error rate (FWER) was used to investigate differences between PPA variants and controls. Pearson bivariate correlation analysis was applied to describe correlations among cortical volumes, tract-specific volumetric measurements, and neuropsychological scores in the patient group.

To determine the variables predicting repetition deficits, we performed a hierarchical multiple linear regression analysis. This method first introduces all selected independent variables (i.e., gray and white matter measures) and the dependent variable (i.e., repetition) in a correlation analysis. Their contribution is then compared against a removal criterion. The variables with the least contribution to the model are subsequently removed and the reduced model is re-estimated for the remaining variables.

Multiple comparison corrections were applied. FDR-controlling procedures provide less stringent control of type I errors compared to FWER-controlling procedures (such as the Bonferroni correction). Thus, FDR-controlling procedures have higher power, at the cost of increased rate of type I errors. We calculated both corrections in our results to fulfill stringent statistical correction while accounting for non-independency in our ROIs. FDR corrections were computed in R (R-project.org).

### Data availability

Anonymized data will be shared on request from any qualified investigator.

## Results

### Behavioral data

Table 1 summarizes the demographic characteristics and the neuropsychological performance of the participants on the repetition subtest and other language tests. Patients with PPA and healthy controls were matched for age, sex, and handedness. Compared to controls, patients with PPA had significantly lower performance in all repetition subtests, including the sentence to word repetition ratio score (table 1). Patients had statistically significant lower speech rate (WPM, MLU) and confrontation object naming (BNT) compared to healthy controls, but no deficits in single word comprehension (PPVT) (table 1).

**Table 1:**
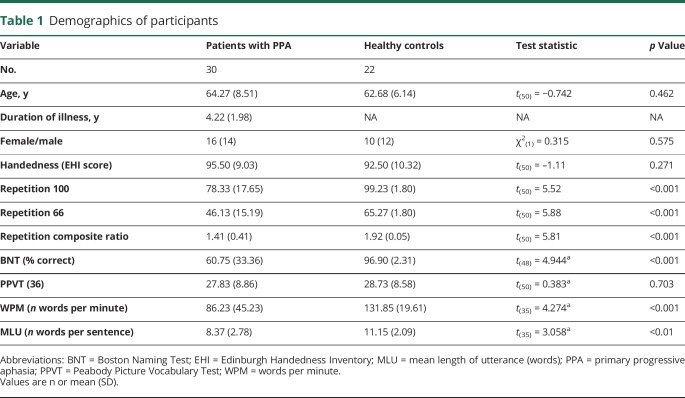
Demographics of participants

### White matter tractography

**Figure 2:**
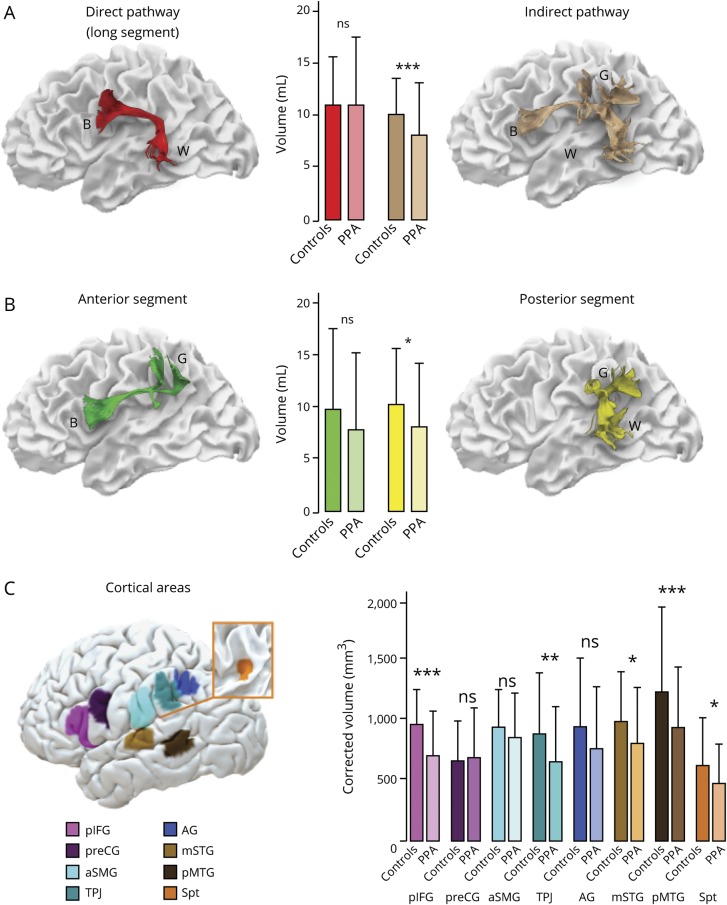
Group differences between patients with primary progressive aphasia (PPA) and controls in the perisylvian white matter and cortical areas (A–C) Error bars indicate 2 SDs from the mean. *Independent *t* test significance level *p* < 0.05. **Independent *t* test significance level *p* < 0.01. ***Independent *t* test significance level *p* < 0.001. AG = angular gyrus; aSMG = anterior supramarginal gyrus; B = Broca territory; G = Geschwind territory; mSTG = middle part of the superior temporal gyrus; ns = not significant; pIFG = posterior inferior frontal gyrus; pMTG = posterior middle temporal gyrus; preCG = precentral gyrus; Spt = Sylvian parietal temporal area; TPJ = temporo-parietal junction; W = Wernicke territory.

Patients with PPA had significantly lower volumes (mL) of the indirect pathway (mean 7.15 ± 2.26) compared to healthy controls (mean 8.89 ± 1.66; *t*_[50]_ = 3.041, *p* < 0.005, d = 0.86) while no significant differences were observed for the direct pathway between patients (mean 9.14 ± 3.04) and controls (mean 9.89 ± 3.05; *t*_[50]_ = 0.204, *p* = 0.836, d = 0.08) (figure 2). When the anterior and posterior segments of the indirect pathway were analyzed separately, differences between patients and healthy controls were statistically significant for the posterior segment (patients: mean 7.24 ± 2.75; controls: mean 9.14 ± 2.47; *t*_[50]_ = 2.56, *p* < 0.05, d = 0.73), while differences in the anterior segment did not reach statistical significance (patients: mean 6.92 ± 3.32; controls: mean 8.64 ± 3.53; *t*_[50]_ = 1.795, *p* = 0.079, d = 0.49) (figure 2).

For the PPA group, the correlation analysis indicated that higher repetition 100 scores were associated with larger measures of the volume of the indirect pathway (*r* = 0.55, *p* < 0.01). Within the indirect pathway, the correlation was driven by the posterior segment (*r* = 0.46, *p* < 0.01) while correlation with the anterior segment did not reach statistical significance (*r* = 0.31, *p* = 0.09). The same analysis was not significant for the long segment of the arcuate fasciculus (*r* = 0.20, *p* = 0.92). Similar results were observed for the sentence-to-word ratio, where reduced volume of the indirect pathway was related to more severe repetition deficits (*r* = 0.47, *p* < 0.01), which was driven by the posterior segment (*r* = 0.41, *p* < 0.05), while correlation with the anterior segment did not reach statistical significance (*r* = 0.28, *p* > 0.1).

No statistically significant correlations were observed between the 3 segments of the arcuate fasciculus and naming (BNT) or single word comprehension (PPVT). Greater deficits in speech rate measures were observed in those patients with more severe damage of the indirect pathway of the arcuate fasciculus (WPM: *r* = 0.536, *p* < 0.01; MLU: *r* = 0.673, *p* < 0.001), especially to its anterior segment (WPM: *r* = 0.531, *p* < 0.01; MLU: *r* = 0.675, *p* < 0.001).

### Gray matter morphometry

Gray matter volume of cortical language areas was statistically different between patients and controls for the posterior inferior frontal gyrus (*t*_[50]_ = 5.13, *p* < 0.001, d = 1.45), superior (*t*_[50]_ = 2.595, *p* < 0.012, d = 0.73) and middle temporal areas (*t*_[50]_ = 3.813, *p* < 0.001, d = 1.08), area Spt (*t*_[50]_ = 2.696, *p* < 0.05, d = 0.76), and temporo-parietal junction (*t*_[50]_ = 2.725, *p* < 0.01, d = 0.77) (figure 2).

**Figure 3:**
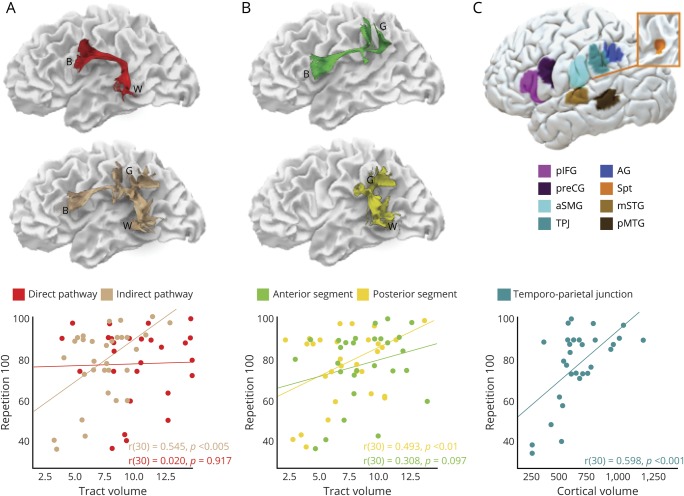
Correlations between repetition and volumetric measurements Correlations between repetition 100 and white matter measures of the direct and indirect pathways (A), the anterior and posterior segments of the arcuate fasciculus separately (B), and the volume of the posterior supramarginal gyrus (C) in patients with primary progressive aphasia. AG = angular gyrus; aSMG = anterior supramarginal gyrus; B = Broca territory; G = Geschwind territory; mSTG = middle part of the superior temporal gyrus; pIFG = posterior inferior frontal gyrus; pMTG = posterior middle temporal gyrus; preCG = precentral gyrus; Spt = Sylvian parietal temporal area; TPJ = temporo-parietal junction; W = Wernicke territory.

In the PPA group, the only statistically significant correlations that survived correction for multiple comparisons were those between the cortex of the temporo-parietal junction and scores in repetition 100 and repetition 66 (table 2 and figure 3).

**Table 2:**
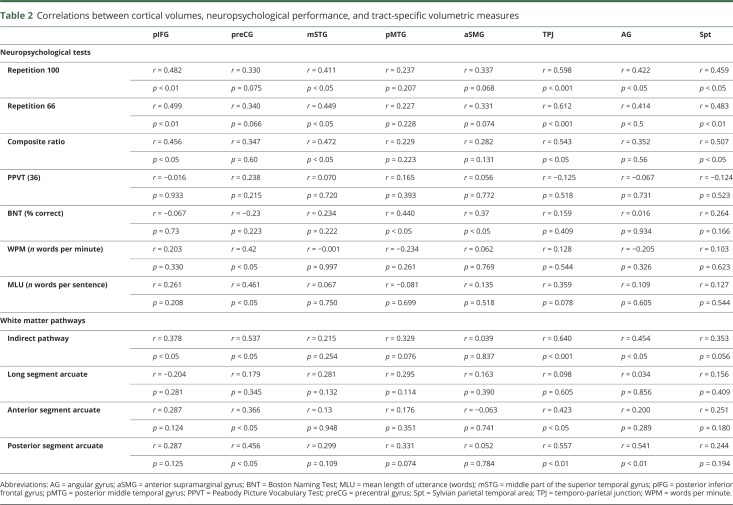
Correlations between cortical volumes, neuropsychological performance, and tract-specific volumetric measures

### Predictive gray matter and white matter contribution to repetition

A hierarchical regression analysis was performed for a first-level model that included only the volume of the indirect pathway of the arcuate fasciculus and a second-level model that included both the volume of the indirect pathway and the cortical volume of the TPJ. The first model indicated the indirect pathway as a statistically significant predictor of repetition deficits (*R*^*2*^ = 0.271, *F*_*1,28*_ = 11.805, *p* = 0.002). The second model showed that the predictive value of the indirect pathway declined (second-level model: *R*^*2*^ = 0.402, *F*_*2,27*_ = 9.088, *p* = 0.001) when the gray matter volume was added to the model (*R*^*2*^ change: *F*_*1,27*_ = 4.778, *p* = 0.038). The indirect pathway (β = 0.545, *t*[3.436], *p* = 0.002) and the TPJ (β = 0.423, *t*[2.186], *p* = 0.038) were independent predictors of repetition deficits. All other variables were automatically excluded from this analysis. The same analysis was conducted to predict naming deficits and identified the middle temporal region as a statistically significant predictor (*R*^*2*^ = 0.445, *F*_*1,27*_ = 6.66, *p* < 0.05). All white matter measures were excluded by the analysis. These results indicate the specificity of our proposed model to repetition deficits in comparison to, for example, confrontation naming.

### Correlations between tractography and gray matter

Overall, there was a general tendency of positive correlation between the volumes of the white matter tracts and the volumes of the selected cortical regions. However, after correcting for multiple comparisons, only the correlation between the TPJ and the volume of the indirect pathway of the arcuate fasciculus remained statistically significant (table 2). No statistically significant correlations were detected between the cortical regions and the volume of the direct pathway (table 2).

**Figure 4:**
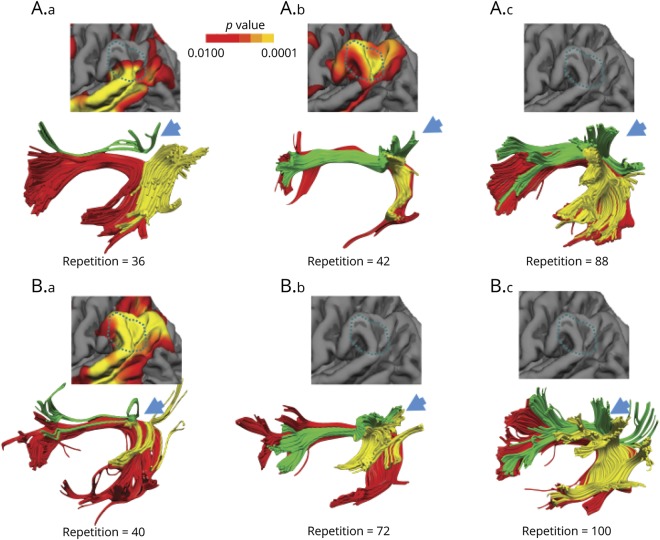
Individual atrophy patterns of the language network and cortical area Reconstruction of the direct (long segment, red) and indirect (anterior segment, green; posterior segment, yellow) pathways of the arcuate fasciculus in some representative patients with primary progressive aphasia (PPA) with different degrees of repetition impairment (repetition = 100 normal repetition). Individual significant atrophy patterns of the temporo-parietal cortex are also shown (warmer colors represent a significant reduction and corresponding *p* values are displayed by the color bar). The blue arrows indicate the cortical projections of the indirect pathway to the TPJ. (A) Three patients with agrammatic PPA variant. (A.a) A patient with severe repetition deficits (repetition = 36) and marked volume loss of the anterior segment of the indirect pathway but preserved posterior segment. The cortical atrophy involves only the most inferior part of the TPJ. (A.b) The patient presented with significant repetition deficits (repetition = 42) but in this case volume loss was more evident for temporo-parietal cortex and the posterior segment. (A.c) The patient presented with mild repetition deficits without detectable evidence of cortical atrophy and white matter volume loss. (B) Three patients with logopenic PPA. (B.a) The patient had severe repetition deficits (repetition = 40) and marked volume loss of both anterior and posterior segments of the indirect pathway. Severe cortical atrophy was also evident in the TPJ for this patient. (B.b) The patient showed moderate repetition deficits associated with reduced volume of both segments of the indirect pathway but no cortical atrophy. (B.c) The patient, who presented with intact repetition, showed no cortical atrophy in the TPJ and normal tract volume of the anterior and posterior segments. Note that the left patients in A and B with normal long segment volume indicate that the atrophy of the indirect pathway is not associated with the severity of repetition impairment.

While the correlation indicates a link between white matter volume loss and cortical atrophy at the group level, the pattern of gray matter and white matter damage in every patient was heterogeneous, as shown in figure 4. The direct pathway represented by the long segment of the arcuate fasciculus does not constitute a valid or alternative route for repetition as it appears of normal size in some patients with severe repetition deficits (see top left nonfluent/agrammatic patient in figure 4A). Likewise, among the same PPA variant, white matter damage along the indirect pathway can be heterogeneous and affect the anterior segment, the posterior segment, or both. The association between repetition deficits and degree of cortical atrophy in the TPJ is also heterogeneous as some patients with less severe atrophy perform worse on repetition tasks compared to others with less atrophy in the same area (see left and middle patients in figure 4A). Similarly, the association between white matter damage and cortical atrophy varies from patient to patient. For example, both cortical atrophy and white matter damage of the indirect pathway are evident in some patients with severe repetition deficits (see left patient in figure 4B) whereas in other patients with severe repetition deficits damage is more evident in the white matter (see left patient in figure 4A) or the cortex (middle patient in figure 4A). Together, our statistical results and these illustrative patients indicate that it is the unique combination of cortical and subcortical pathology that is responsible for the emergence of repetition deficits irrespective of the clinical PPA subtype.

## Discussion

**Figure 5:**
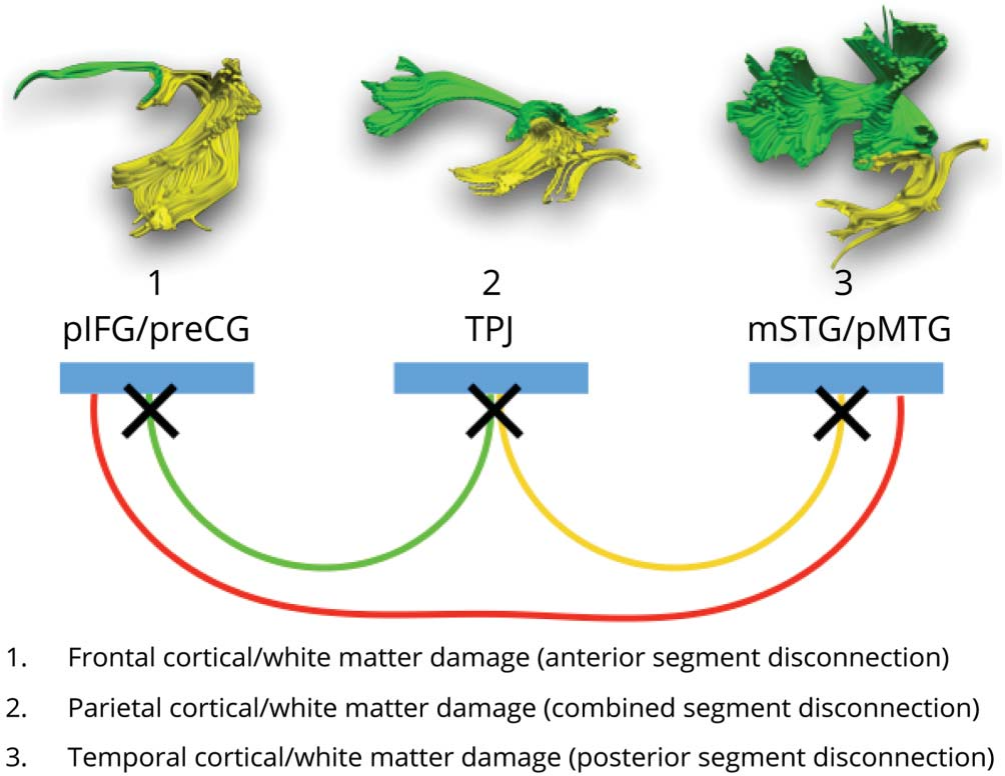
A revised anatomical model of repetition The colors indicate the posterior (yellow) and anterior (green) segments of the arcuate fasciculus forming the indirect pathway for repetition, and the long segment of the direct pathway (red). Temporal, parietal, and frontal perisylvian language areas indicated in blue. The crosses are putative locations of lesions associated with repetition deficits and conduction aphasia. mSTG = middle part of the superior temporal gyrus; pIFG = posterior inferior frontal gyrus; pMTG = posterior middle temporal gyrus; preCG = precentral gyrus; TPJ = temporo-parietal junction.

In this study, we reported that atrophy of both temporo-parietal cortex and white matter of the indirect pathway of the arcuate fasciculus is more prominent in those patients with PPA with severe repetition deficits. The same correlation was not detected for the direct connections between Wernicke and Broca territories. Our findings reconcile the controversy surrounding the manifestation of conduction aphasia as they suggest that both cortical and subcortical lesions may contribute to repetition deficits if the damage affects the indirect pathway of the arcuate fasciculus or its cortical terminations in the TPJ. We therefore suggest a revision of the classical model for repetition that takes into account the cortex of the TPJ and its connections to both Wernicke and Broca territories. Our revised model provides an anatomical framework for interpreting the spectrum of neuropsychological presentations of patients with repetition deficits and the associated variability in lesion location (figure 5).

Particularly relevant to our study is a previous neuropsychological model for repetition that theoretically distinguishes patients with repetition deficits according to deficits in retaining or producing an ordered set of words.^26^ This model indicates that in patients with an impaired ability to retain words (true repetition syndrome), the primary deficit is in the working memory route, whereas patients with impaired reproduction (reproduction syndrome) have intact working memory, but they fail to repeat infrequent multisyllabic words correctly. According to our indirect pathway framework, we argue that in the former group damage is localized to the posterior segment of the arcuate fasciculus and its cortical terminations, whereas in the latter group, damage is affecting the anterior segment and its cortical areas. Patients with mixed presentation of deficits are more likely to have damage to the inferior parietal cortex or both anterior and posterior segments (figure 5). Indeed, the statistically significant positive correlation between the volume of the posterior segment of the arcuate and the sentence-to-word ratio, which is sensitive to working memory deficits, supports these predictions. In future studies, assessment of verbal and nonverbal working memory and sequencing should be included to further separate the contribution of each process to repetition in relation to the anterior and posterior segments composing the indirect network model.

The results of our cortical morphometry analysis indicated that within the posterior perisylvian regions the temporo-parietal cortex was highly correlated with repetition deficits in PPA. Historically, the inferior parietal cortex has been indicated to contain the cognitive module necessary for several language functions, including repetition.^27^ This hypothesis is further supported by subsequent imaging studies, which consistently link repetition deficits with lesions affecting the temporo-parietal cortex.^8,28–32^ In recent years, the use of quantitative lesion mapping has indicated that the cortical damage most frequently associated with repetition deficits is indeed located within the left temporo-parietal region,^8,22,23^ although the exact anatomical definition of the relevant area remains controversial. Some previous lesion-based studies, for example, located the region for repetition in the anterior supramarginal gyrus,^22^ whereas others reported a more posterior location in the inferior parietal lobule (i.e., posterior supramarginal gyrus and angular gyrus) and superior temporal gyrus.^8,23^ Voxel-based morphometry studies in PPA reported a correlation between repetition deficits and gray matter volume in the posterior superior temporal gyrus and supramarginal gyrus.^8,12,23^ Similarly, findings from functional imaging studies in healthy controls indicate activation of a temporo-parietal region during repetition tasks,^33,34^ which includes the Sylvian parieto-temporal area (Spt) buried in the most caudal depth of the lateral fissure. Collectively, these findings suggest an indirect route for repetition from Wernicke to Broca region relaying in the temporo-parietal cortex.

In our study, all areas previously indicated as important for word repetition were included in the analysis. Thus, we were able to identify the temporo-parietal junction as the area highly associated with repetition deficits. This region lies posterior to the critical region identified using combined perfusion and structural MRI in stroke patients with conduction aphasia.^22^ One possible explanation for this difference is that in this stroke sample, the lesions extended into the white matter of the anterior segment of the arcuate fasciculus, which runs underneath the supramarginal gyrus. According to our model, such lesions can cause repetition deficits through a disconnection of the anterior segment and manifest with a typical reproduction syndrome.^26^

The cortex of the TPJ is in the proximity of Spt but clearly distinct from it, as shown in figure 1. Although the cortical volume of Spt was reduced in patients with PPA, correlations between Spt atrophy and severity of repetition deficits were weak. The observed differences between previous studies of Spt and the current study may be related to methodologic aspects or differences in the populations studied. For example, functional imaging studies provide only group-level peak activations, whereas Spt activation is highly variable and often extending towards the more lateral inferior parietal lobule.^35,36^ Conversely, cortical volume measurements provide only an indication of the cortical areas involved in specific tasks and may be highly dependent on the studied cohort, in our case a group of patients with a heterogeneous neurodegenerative disorder.^2^ This does not necessarily exclude the possibility of the TPJ and Spt sharing a similar function in auditory-motor transformations of speech sounds. Activation studies using a humming of melodic stimuli tasks suggest that Spt may contain distinct populations of cells, some of which are sensory-weighted while others are motor-weighted.^24^ The activity of this area may also not be specific to speech but involve auditory-motor transforms of nonverbal sounds^24^ or gestures,^37^ whereas the more posterior temporo-parietal cortex may have a specific role in language. This interpretation is in line with studies in conduction aphasia stroke patients reporting a location of the areas of maximum lesion overlap in the posterior temporal and inferior parietal lobe^22,23^ and comparative cytoarchitectonic studies pointing at the uniqueness of the human posterior supramarginal cortex (area PFm).^38^ The emergence of a uniquely human cortical area in the TPJ could also be related to the development of far higher capacity for auditory memory in humans. The connections between the posterior supramarginal gyrus and the temporal lobe may, therefore, represent the anatomical substrate for the human ability to maintain words in the phonologic buffer and to repeat them.^39–41^According to our model, lesions to these connections typically manifest with the inability to retain words (i.e., true repetition syndrome).^26^

PPA offers a unique opportunity to perform complementary analysis of the effects of cortical and white matter pathology on language networks.^42–44^ The finding of a direct correlation between cortical morphometry, tractography, and clinical symptoms is in line with postmortem studies demonstrating that in PPA cortical language areas and their connections are both affected. At the same time, the progressive nature of PPA pathologies does not completely alter the cortical and white matter architecture and, compared to stroke, this represents an advantage for studying clinical–anatomical correlations.^45^ It is also important to recognize that the pathology of PPA is heterogeneous, ranging from Alzheimer disease–like pathology to frontotemporal lobar degeneration.^7–10,45^ These disorders affect the cortical structure and the underlying white matter differently, leading to diverse clinical presentations and atrophy profiles.^46^ Indeed, we observed a high variability of anatomical damage with a range of severity within the same PPA subtypes as shown in figure 4. Since there is no absolute one-to-one relationship between the clinical PPA subtype and the neuropathology classification, it is unclear if the interindividual differences are due to different disease processes, disease severity, or other factors.

Finally, the model has important clinical implications as it can be generalized beyond patients with PPA and stroke to include a broader spectrum of language disorders and might be particularly relevant for functional mapping in neurosurgery or language recovery studies. Currently, repetition tasks are rarely used for direct cortical stimulations of the inferior parietal lobe due to the absence of the indirect pathway in the classical model of repetition. Nevertheless, previous case reports in patients with tumor undergoing intraoperative functional mapping and preliminary evidence from electrocorticography in patients with epilepsy suggest that the inferior parietal lobe is an obligatory route for repetition.^47^ We hope that the use of repetition tasks in patients with tumor or epilepsy in the temporo-parietal region will help to validate our model and minimize language impairment in these patients. Likewise, the efficacy of stimulation therapies based on repetitive transcranial magnetic stimulation would benefit from an exact localization of cortical target regions to foster positive effects and account for interindividual variability.^48^ There are also preliminary reports on the potential benefits of invasive surface electrode^49^ or deep brain^47^ stimulation in patients with language and motor deficits after stroke. The use of diffusion imaging in medical training and its availability in most clinical MRI scanners will guarantee a large application of tractography-derived models of language for clinical teaching and individualized approaches to patient care.

While this study had the advantage of using an integrative multimodal imaging approach, several limitations should be acknowledged. The total study cohort was of a reasonable size to perform these analyses, but the stratification of PPA variants was small and underlying neuropathology was unknown for most. We therefore refrained from performing a separate analysis for the subgroups or underlying neuropathology, although this approach would undoubtedly lead to valuable insight on the relationship among the nature of the underlying pathology, the individual clinical profile, and the disease progression, especially if longitudinal data and postmortem examination were available. Future studies that are combining PET with structural analysis and diffusion tractography may indeed reveal a white matter vulnerability of specific tracts towards different underlying pathologies associated with PPA variants. Also, while our study indicates an indirect route for repetition, we cannot exclude that the direct pathway may also have a role in other forms of repetition that were not assessed in our study. For example, in a previous investigation, we demonstrated that the direct pathway has a significant role in learning pseudo-words.^50^ Learning pseudo-words is associated with activation in the Wernicke and Broca regions and is facilitated by subvocal repetition through the direct pathway of the arcuate fasciculus.^50^ Finally, we recognize that the tractography analysis performed was based on a simplified model of the direct and indirect networks. Both direct and indirect networks project to multiple cortical regions within the inferior parietal lobule, suggesting the possibility of further segregations. With increased spatial resolution and faster acquisitions, future studies may be able to analyze different connections to each inferior parietal region and reveal functional specialization within the indirect network.

Our study benefitted from a combined cortical and subcortical imaging approach to demonstrate that repetition deficits in PPA are associated with damage to an indirect pathway connecting temporal and frontal regions via the parietal lobe. These findings indicate a possible functional segregation within the arcuate fasciculus and the temporo-parietal cortex. The indirect pathway could play a pivotal role in phonemic-motor transforms and auditory short-term memory storage of speech sounds that are necessary for repetition. Both the temporo-parietal cortex and the anterior and posterior segments, which form the indirect pathway of the arcuate fasciculus, should be included in future revisions of current language networks for educational and clinical purposes.

## Glossary

BNT: Boston Naming Test
eTIV: estimated total intracranial volume
FDR: false discovery rate
FsAverage eTIV: the estimated total intracranial volume for the FreeSurfer average participant
FWER: familywise error rate
MLU: mean length of utterance
PPA: primary progressive aphasia
PPVT: Peabody Picture Vocabulary Test
ROI: region of interest
Spt: Sylvian parietal temporal area
TPJ: temporo-parietal junction
WPM: words per minute
